# Medical College of the State of South Carolina

**Published:** 1857-04

**Authors:** 


### MEDICAL COLLEGE OF THE STATE OF SOUTH CAROLINA.

We have received the Catalogue of the Medical College of the State of South Carolina, in Charleston, for the Session of 1856–7, from which we learn that they numbered in their class 245; we take great pleasure in recording the prosperous condition of the Institution.

